# CHI3L1 in Multiple Sclerosis—From Bench to Clinic

**DOI:** 10.3390/cells13242086

**Published:** 2024-12-17

**Authors:** Izabela Jatczak-Pawlik, Anna Jurewicz, Małgorzata Domowicz, Alicja Ewiak-Paszyńska, Mariusz Stasiołek

**Affiliations:** Department of Neurology, Medical University of Lodz, Kosciuszki Street 4, 90-419 Lodz, Poland; izabela.jatczak@umed.lodz.pl (I.J.-P.); anna.jurewicz@umed.lodz.pl (A.J.); malgorzata.domowicz@umed.lodz.pl (M.D.); alicja.ewiak-paszynska@umed.lodz.pl (A.E.-P.)

**Keywords:** multiple sclerosis, chitinase-3-like 1 protein, biomarkers, cerebrospinal fluid

## Abstract

Multiple sclerosis (MS) is a chronic demyelinating disease of the central nervous system (CNS) with a complex and not fully understood etiopathological background involving inflammatory and neurodegenerative processes. CHI3L1 has been implicated in pathological conditions such as inflammation, injury, and neurodegeneration, and is likely to play a role in the physiological development of the CNS. CHI3L1 is primarily produced by CNS macrophages, microglia, and activated astrocytes. The CHI3L1 expression pattern in MS lesions might support the important role of astrocytes in modulating inflammatory processes in this disease. The potential applications of CHI3L1 as a biomarker in MS are multifactorial. The measurement of CHI3L1 in body fluids might find its role in the early diagnosis of MS. In further stages, the monitoring of CHI3L1 levels might provide information on disease severity and progression, enabling a better adjustment of therapeutic strategies. Importantly, CHI3L1 might potentially serve as a marker of ongoing glial activation, reflecting the dynamic response of the CNS cells to the inflammatory processes in MS. Although preliminary findings have been promising, further research is needed to validate the utility of CHI3L1 measurements in the diagnosis and prediction of the progression of MS. Additionally, comparisons with other biomarkers might be useful in clinical practice.

## 1. Characteristics of Multiple Sclerosis (MS)

Multiple sclerosis (MS) is a chronic demyelinating autoimmune disease of the central nervous system (CNS) characterized by three main pathological processes: inflammation, astrogliosis, and neurodegeneration. These processes lead to multifocal demyelination and damage to axons in the brain and spinal cord, which results in potentially severe clinical impairments [1]. The etiology of MS remains not sufficiently understood. However, based on available data, it is believed that the pathogenesis is multifactorial, and involves genetic, immunopathological, and environmental factors. Profound genome-wide association studies identified numerous gene regions associated with increased or reduced disease susceptibility; however, each of the individual genes provides only a minor effect. Most of these genes are involved in immunological processes, which might confirm the immunological background of MS. The current research is also focused on the interactions between environmental factors and genetic predisposition for MS development. The known environmental factors affecting MS course include tobacco use, vitamin D deficiency, obesity, and Epstein–Barr virus infection [2,3]. It is estimated that 2.3 million people in the world are affected by MS [4]. MS develops mostly in young adults, with the peak of disease onset between 20 and 40 years of age for relapsing-remitting MS (RRMS), and shows evident female predominance (female: male ratio 3:1) [5]. For primary progressive MS (PPMS), the age of onset is typically in the fifth decade of life, with less evident female predominance [6].

The commonly accepted current phenotypic classification of MS recognizes RRMS, clinically isolated syndrome (CIS), radiologically isolated syndrome (RIS—MS-like lesions on magnetic resonance imaging [MRI] without clinical manifestation), primary progressive MS (PPMS), and secondary progressive MS (SPMS) [7]. The 2010 McDonald criteria of MS introduced the term clinically isolated syndrome (CIS) for the first episode of neurological symptoms lasting at least 24 h, suggesting MS. The 2017 update of McDonald criteria [8] allows for an MS diagnosis in patients with CIS if the dissemination in time (DIT) (e.g., oligoclonal bands in MRI data) and dissemination in space (DIS) (e.g., MRI data) are confirmed.

MS is believed to be an autoimmune disease with the pathologic activation of autoreactive lymphocytes by cross-reactivity with foreign antigens or by recognizing CNS antigens leaking into deep cervical lymph nodes [9]. T lymphocytes are implicated as one of the most important players in these processes. The importance of CD4+ T lymphocytes is supported by the identification of human leukocyte antigen (HLA class II) genes as a major genetic risk factor for MS [10]. Among CD4+T cells, proinflammatory T helper 1 (Th1) secreting interferon-gamma (IFN-γ), interleukin (IL)-2, and tumor necrosis factor alpha/beta (TNF-α/β) and T helper 17 (Th17) secreting IL-17 were linked to relapses of MS [11,12]. On the other hand, regulatory T lymphocytes (Tregs and Tr1) were connected to the remission and downregulation of immune processes in MS [13]. The role of CD8+ T lymphocytes was indicated by the fact that they are the most abundant T cell type in MS lesions, and by the identification of HLA class I genes as a genetic risk factor for MS. The B lymphocytes, other important cells in MS, have been described to have several functions in immune processes in MS, including antigen presentation to T lymphocytes as antigen-presenting cells (APC) and the production of proinflammatory and anti-inflammatory cytokines. The role of regulatory B lymphocytes, a subset of B cells, is the adjustment of the immune response both in the periphery and in the CNS, which potentially protects the CNS against tissue damage. Additionally, B lymphocytes might differentiate into plasma cells and produce autoantibodies participating in antibody-dependent cellular cytotoxicity (ADCC). The clonal expansion of B cells in CSF is suggested to be responsible for the intrathecal production of oligoclonal bands in MS [14]. What is important therapies based on B cell depletion are some of the most effective in MS [15]. Microglia are CNS-restricted immune cells that can be polarized into M1 proinflammatory profile (increasing CNS destruction and immune activation) and M2 profile involved in myelin debris clearance, enabling remyelination. Macrophage infiltration was described in the CNS inflammatory sites in MS, and these cells might be polarized into M1 and M2 profiles as microglia. Another type of CNS-restricted cells are astrocytes, which are glial cells, not strictly immune cells, which might modulate inflammatory processes in CNS via the secretion of cytokines, chemokines, and cell surface ligand expression. The less abundant immune cells, such as dendritic cells (DC), gamma-delta T lymphocytes, natural killer (NK) cells, mucosal-associated invariant T cells, and mast cells, have also been shown to play a role in MS [16,17]. The onset of MS is believed to be triggered by the peripheral proinflammatory activation of autoreactive T lymphocytes, which then cross the blood–brain barrier (BBB), engaging adhesion molecules and metalloproteinases (MMPs). After crossing the BBB, lymphocytes are reactivated by APC (dendritic cells, microglia, B lymphocytes) in the CNS, causing its clonal expansion. The cytokines secreted by clonally expanded lymphocytes activate macrophages, microglia, DCs, and astrocytes, and aggravate CNS demyelination and destruction [18].

Recently, the role of previously underappreciated cells—neutrophils—in MS pathology has been investigated [19,20]. Although their exact involvement is unclear, neutrophils might enhance the disease course by autoimmune cell activation in the periphery, but not by direct involvement in the local CNS pathology [21]. In consequence, these immunological processes localize in the CNS (augmented in the periphery), leading to chronic neurodegeneration associated with microglial activation, astrogliosis, and persistent inflammation [22].

The complex etiopathology, the wide spectrum of clinical presentation, and the lack of disease-specific markers might make the diagnostic process challenging, often leading to significant delays or misdiagnoses [23,24,25]. Magnetic resonance imaging (MRI) is a crucial diagnostic tool in MS. However, even “typical” MS lesions on the MRI of CNS result from other diseases, and, thus, careful differential diagnosis is required. The high number and volume of focal lesions and the extent of brain atrophy on MRI have been linked to an increased risk of disability in MS patients [24]. Still, the correlation between classical MRI parameters, laboratory findings, patient’s actual symptoms, and disease progression is not always satisfactory [26]. Thus, current limitations require the identification of biomarkers enabling early and unambiguous diagnosis, predicting a future disease activity as well as allowing for the efficient monitoring of disease course, therapeutic response, and identifying the risk of side effects in individual patients [27]. Among potential biomarkers, molecular factors, which are possible to be measured in body fluids, particularly in peripheral blood but also in cerebrospinal fluid (CSF), have been considered the most promising. Currently, the MS prognostic biomarkers that have been investigated the most include IgG oligoclonal bands [28], neurofilament light chain (NfL) [29,30,31], glial fibrillary acidic protein (GFAP) [30,31], chitinase 3-like protein 1 (CHI3L1) in CSF [32], and serum [33,34]. The CHI3L1 secretory glycoprotein emerges as one of the most promising candidates for a biomarker of clinical diagnosis and prognosis in MS. However, it has to be underlined that the protein is not specific for MS (see below), and its potential use as a biomarker has to always be put in an appropriate clinical context.

## 2. CHI3L1 as a Potential Biomarker for MS

CHI3L1, known as YKL-40 in humans and breast regression protein 39 (BRP-39) in mice [35], is a secreted glycoprotein encoded by the CHI3L1 gene composed of 10 exons located on chromosome 1q32.1. This 40 kDa protein was initially discovered in cultured chondrocytes and synovial cells and, subsequently, in other cell types [36]. CHI3L1 belongs to the chitinase family (glycosyl hydrolase family 18, GH18), and to a subgroup of chitinase-like protein (CLP) [37] lacking enzymatic activity but still exhibiting high affinity binding to chitin [36,38]. This suggests that CHI3L1 plays an important non-enzymatic biological role by binding to several receptors [39]. CHI3L1 is essential for protecting against pathogens, injury response, inflammation, apoptosis, inflammasome activation, Th1/Th2 balance, M2 macrophage differentiation, and tissue repair via the regulation of oxidant injury [40]. Though chitin [41] itself is absent in mammals (found in fungi, insects, etc.), mammalian chitinases, including CHI3L1, are present in several tissues/organs including skin, intestine, joints, and lungs [42,43]. CHI3L1 is synthesized and secreted by several types of cells, including synoviocytes, chondrocytes, fibroblast-like cells, and tumor cells [44]. In the CNS under inflammatory conditions (e.g., MS), CHI3L1 is produced by infiltrating immune cells (e.g., macrophages and neutrophils), endothelial cells, microglia, and astrocytes, rendering it a potential biomarker [45,46].

Several receptors for CHI3L1 have been described, including receptors for advanced glycation end product (RAGE), CD44, interleukin-13 receptor subunit alpha-2 (IL-13Rα2), chemoattractant receptor-homologous molecule expressed on Th2 cells (CRTH2), galectin-3 (Gal-3), and transmembrane protein 219 (TMEM219), also known as the insulin-like growth-factor-binding protein 3 receptor (IGFBP3R). CHI3L1 binding to its receptor determines its role in inflammation, cancer, and neurodegenerative disorders.

RAGE is expressed on various cell types including vascular endothelial cells, immune cells (monocytes/macrophages, dendritic cells), neurons, and epithelial and cardiac cells [47,48,49,50,51]. The expression of RAGE might be upregulated in response to various stimuli, including inflammation, oxidative stress, and aging [52]. CHI3L1 binds to RAGE, activating multiple signaling pathways promoting cell growth, inflammation, and apoptosis [53]. RAGE uniquely activates the nuclear factor kappa-light-chain-enhancer of activated B cells (NF-κB), creating a positive feedback loop that exacerbates inflammation [53]. In neurodegenerative diseases, CHI3L1-RAGE interactions influence neurogenesis by affecting neural stem cell proliferation and differentiation [54,55]. CD44 is particularly expressed on the surface of activated lymphocytes and tumor cells [56], and its activation promotes cell proliferation, cancer cell growth, and metastasis. The CHI3L1 binding to CD44 activates several signaling pathways including mitogen-activated protein kinases/extracellular-signal-regulated kinases (MAPK/ERK, AKT/PKB), and Wnt/β-catenin signaling involved in neoplastic progression, which makes it a potential therapeutic target in cancers such as gastric cancer [57] and glioblastoma [58].

The abundant expression of IL-13Rα2 is primarily detected in various types of cancer, including glioblastoma, colorectal, and breast cancer [59]. CHI3L1 forms a complex with IL-13Rα2 and IL-13, activating several signaling pathways (MAPK/ERK, AKT/PKB, and Wnt/β-catenin) engaged in the regulation of numerous cellular processes such as apoptosis, inflammation, immune response, melanoma metastasis, and transforming growth factor β1 (TGF-β1) production [60]. N-glycosylation of CHI3L1 is crucial for its binding to IL-13Rα2, highlighting the importance of post-translational modifications in this interaction [60].

Additionally, in the absence of IL-13Rα2, CHI3L1 binds with CRTH2, which promotes fibrotic responses and tissue damage [61] and impairs oligodendrocyte precursor functioning, potentially contributing to neurodegenerative processes [62]. CRTH2 is predominantly expressed in immune cells, particularly eosinophils, basophils, Th2 cells, innate lymphoid cells type 2 (ILC2), mast cells, dendritic cells, and monocytes/macrophages [63,64].

Gal-3 is expressed in various tissues and cell types such as immune cells (macrophages, T and B cells, and dendritic cells), epithelial cells, endothelial cells, fibroblasts, and cancer cells [65]. This receptor interacts with CHI3L1 and IL-13Rα2, forming a complex that activates the Wnt/β-catenin signaling pathway [66]. Depending on its cellular location, Gal-3 can either promote apoptosis or induce macrophage differentiation and tissue repair [66].

TMEM219 is primarily expressed in pancreatic cells [67] and enhances IL-13 binding to the IL-13Rα2-CHI3L1 complex, activating MAPK/ERK and protein kinase B (AKT/PKB) signaling pathways [68]. This interaction regulates apoptosis, oxidative stress, and tumor metastasis [68].

The CHI3L1 has been found to be involved in neurogenesis and physiological CNS development. On the contrary, in adult CNS, its presence is associated with pathological conditions [69,70,71], including MS. Several studies reported the presence of CHI3L1 within white matter lesions of MS patients, specifically localized in astrocytes and macrophages/microglia (CD68 positive cells) [69,70,71]. Pinteac et al. highlighted the involvement of protoplasmic astrocytes in CHI3L1 production in active MS lesions and other neurodegenerative diseases [69]. Macrophages, but not T lymphocytes (CD3+ cells), present in the perivascular spaces in the CNS-expressed CHI3L1 [70,71]. The abundant expression of CHI3L1 was detected in reactive astrocytes and macrophages in active inflammatory MS lesions, whereas in low-active or non-active lesions, the expression of CHI3L1 in these cells was negligible [33,70,72]. These findings might confirm the role of CHI3L1 as a marker of activated astrocytes in MS [45,73,74,75,76]. Astrocyte activation, one of the hallmarks of MS, contributes to autoimmune responses and neuronal damage, demyelination, and axonal degeneration [77].

Moreover, several studies have found that the level of CHI3L1 in CSF correlated with disease activity. It has been suggested that CHI3L1 detected in the CSF had been predominantly produced within the CNS rather than transferred from the blood, as Canto et al. have shown that CHI3L1 has a limited ability to cross the BBB [70]. The concentration of CHI3L1 in the CSF of MS patients was six times higher than in serum [78], indicating its local CNS production. The astrocytes within active and chronic active lesions are considered the most important producers of CHI3L1 in the CNS, as determined by the in situ hybridization and immunohistochemistry studies of MS brains [45,46,71]. Moreover, in vitro studies have demonstrated that the proinflammatory stimulation of astrocytes induced increased CHI3L1 release [79,80], confirming the CNS origin of CHI3L1 in MS patients. The CD14 ^low^ cells, isolated from CSF, are unlikely to migrate into the CNS due to a lack of CCR5 receptors [81,82], and have been found to express CHI3L1. Canto et al. have proposed that the expression of CHI3L1 in CD14 ^low^ monocytes as well as in microglia might be responsible for the basal low-level CNS expression of CHI3L1 unrelated to inflammation [70]. Under inflammatory conditions, other than macrophages/microglia and CD14 ^low^ monocytes, CNS cells like astrocytes are suggested to secrete an increased amount of CHI3L1 [70].

In vitro studies demonstrated that macrophages release CHI3L1 and several cytokines, which may induce CHI3L1 production by astrocytes. Accordingly, astrocytes, localized near microglia, have been suggested as the primary source of CHI3L1 [45]. Bhardwaj et al. identified a specific pathway involving IL-1, signal transducer and activator of transcription 3 (STAT3), and RelB/p50 complexes that induce CHI3L1 expression in astrocytes during inflammation [83].

The increased level of CHI3L1 in the serum of MS patients [5,34,71,84] might result from the secretion of this glycoprotein by several cell types, including neutrophils [85], macrophages [38,86], endothelial cells [87], and smooth muscle cells [88]. Neutrophils appear to significantly contribute to serum CHI3L1 levels during inflammation [85]. This hypothesis might be supported by studies showing elevated CHI3L1 levels in neutrophils isolated from patients with inflammatory diseases [89,90,91]. However, further studies are needed to fully understand the source of increased serum CHI3L1 levels in MS.

Enzyme-linked immunosorbent assay (ELISA) and single molecule array (SIMOA) have been used to measure CHI3L1 levels in body fluids. While ELISA is widely used and easily available, SIMOA offers superior sensitivity, as demonstrated by Disanto et al. [29]. For daily routine, ELISA remains a suitable method for detecting CHI3L1, particularly in CSF, where levels are detectable even with less sensitive techniques.

### 2.1. CHI3L1 as an Early MS Biomarker

Several studies have investigated the role of CHI3L1 in the early stages of MS. Fino et al. demonstrated significantly higher CSF CHI3L1 levels in CIS and RRMS patients than in controls [55], suggesting the involvement of CHL3L1 in the pathogenesis of MS. Thouvenot et al. found significantly higher CSF CHI3L1 levels in RRMS compared to RIS patients, suggesting a potential association between CHI3L1 levels and disease severity [92]. Moreover, CSF CHI3L1 levels were higher in RIS patients with positive CSF (presence of oligoclonal bands and/or an elevated IgG index) than those with negative CSF, indicating a possible link to disease progression [92]. Several studies have also shown that CIS patients with higher CSF CHI3L1 levels faster convert to clinically definite MS, suggesting that CHI3L1 might be a prognostic biomarker for disease activity and disability progression in the early stages of multiple sclerosis [70,71,73,78,92,93]. In accordance with previous studies, Kušnierová et al. reported higher levels of CSF CHI3L1 in MS patients compared to CIS patients [94]. Importantly, in both these groups (CIS and MS), CSF CHI3L1 levels were higher than in controls. All these data suggest that CHI3L1 might be a good candidate for the assessment of disease activity. However, Matute-Blanch et al. did not find a correlation between CSF CHI3L1 levels and conversion to CIS [95]. The small amount of data available on RIS and CHI3L1 concentrations might limit the statistical power of such analyses.

Elevated CSF [70,93,96] and serum [34] CHI3L1 levels in patients with CIS and RRMS have been associated with disability progression assessed by the expanded disability status scale (EDSS). Similarly, higher CSF CHI3L1 levels have been correlated with the number of brain lesions on MRI in CIS patients [70,78], as well as with brain volume loss in CIS and RRMS patients [97]. However, Stoop et al. suggested that upregulated CHI3L1 levels might not be a specific marker for inflammatory processes in MS correlated with disease activity and progression, but might be the result of inflammation in CNS irrespective of disease [98]. In accordance with this idea, elevated levels of CHI3L1 have been observed in a variety of neurological disorders, such as traumatic brain injury, ischemic stroke, Alzheimer’s disease, Parkinson’s disease, amyotrophic lateral sclerosis (ALS), and glioma [99,100,101]. Additionally, CHI3L1 has been associated with various cancers and rheumatoid arthritis, confirming its role as an unspecific inflammatory marker [39,102,103].

Despite the fact that CHI3L1 in CSF might generally correlate with inflammation, it still might be a useful biomarker for identifying disease activity in the early stages of MS and for monitoring disease progression.

### 2.2. CHI3L1 in MS Progression

The correlation between CHI3L1 levels and MS progression remains complex and multifactorial. While numerous studies have demonstrated a significant association between elevated CSF and serum CHI3L1 levels and increased disease severity, especially in progressive forms of MS, some findings have been contradictory.

Several studies have shown that higher CSF and serum CHI3L1 levels are correlated with the degree of disability and cognitive impairment in patients with progressive MS (PPMS and SPMS) compared to control patients [5,32,73,84,96,104,105,106]. Based on these data, CHI3L1 has been suggested as a biomarker for MS progression and severity. Some studies have shown that higher CSF [5,73] and serum [5] CHI3L1 concentrations might predict a more severe course of MS, especially SPMS. These findings indicate the potential role of CHI3L1 in assessing neuroinflammation and monitoring disease progression in MS patients, particularly those with progressive forms. Burman et al. found that CSF CHI3L1 levels were associated with disease activity in RRMS, suggesting its potential role as a biomarker for predicting disease progression in RRMS [72]. Lucchini et al., and Malmeström et al., further support the idea of CHI3L1 involvement in MS pathogenesis, as its elevated CSF levels were associated with a higher risk for relapse, higher activity of disease assessed by MRI, and increased disability progression in RRMS patients [76,107]. In accordance with these data, Correale and Fiol suggest the importance of CHI3L1 levels in CSF for the prediction of RRMS to SPMS conversion [33].

However, not all conducted studies found an association between CSF CHI3L1 levels and MS progression. Sellebjerg et al. did not find a correlation between CSF CHI3L1 levels and the risk of conversion to SPMS or disability progression [108], but found a correlation between elevated CSF CHI3L1 levels and an increased risk of relapses in patients with RRMS [108].

In conclusion, although the existing data suggest a correlation between CSF and serum CHI3L1 levels and MS progression, especially in progressive forms, more studies are necessary to fully comprehend the relationship between this biomarker and disease severity, activity, and progression. Further studies are essential to evaluate the potential utility of CHI3L1 as a biomarker for MS progression.

### 2.3. CHI3L1 Level in the Serum of MS Patients

CSF has been extensively studied as the best source of potential biomarkers for CNS diseases, including MS. However, peripheral blood, despite its “distance” from CNS, is more useful for repeated measurements because of its effortless collection compared to CSF. Taking this into consideration, serum CHI3L1 levels represent an interesting option in the context of repeated measurements to assess MS activity and progression.

Elevated levels of CHI3L1 were found in both the CSF and serum of MS patients (including CIS, RRMS, PPMS, and SPMS), suggesting that serum CHI3L1 levels might be used instead of CSF CHI3L1 levels as a potential diagnostic biomarker [71]. Cantó et al. showed that serum CHI3L1 levels are significantly higher in SPMS and PPMS patients compared to RRMS and healthy controls [84]. Also, Huss et al. and Varhaug et al. found that serum CHI3L1 levels were higher in PMS patients than those with RRMS [5,109]. Additionally, Huss et al. observed a correlation between serum CHI3L1 levels and disease severity in PMS [5]. These data indicate that serum levels of CHI3L1 might be a valuable marker for assessing disease progression [78]. Comabella et al. found that higher CSF levels of CHI3L1 in CIS patients predicted the conversion to clinically definite MS (CDMS). However, such a correlation has not been observed between serum CHI3L1 levels and conversion from CIS to CDMS, as CHI3L1 levels in serum for both CIS patients (converted and non-converted to MS) were similar to each other and significantly higher than controls [78]. Dönder and Ozdemir also found that CHI3L1 serum levels were higher in CIS patients than in MS patients, but the levels did not predict the conversion to CDMS [34].

However, some studies have not confirmed a correlation between the higher CHI3L1 in serum and MS clinical course [32,33,105,110], including studies by Håkansson et al., who showed the limitations of serum levels of CHI3L1 as a biomarker [97]. While CSF CHI3L1 levels were correlated with disease activity, particularly brain volume loss, serum CHI3L1 levels showed no correlation with disease activity [97].

CHI3L1 appears to be a promising non-invasive biomarker for MS. While its levels have been elevated in both CSF and peripheral blood, serum CHI3L1 levels seem more useful for assessing disease progression and activity. However, conflicting data require further study to determine the role of CHL3L1 as a diagnostic and prognostic marker of MS.

### 2.4. Disease-Modifying Therapies (DMT) Influence CHI3L1 Levels in MS Patients

Several disease-modifying therapies (DMT), including IFNβ [109] natalizumab [74,76,98,109,111], mitoxantrone [76], daclizumab [106], and fingolimod [75], have been shown to significantly reduce CSF CHI3L1 levels compared to untreated patients across all MS clinical types (Table 1). This observation could suggest a potential correlation between CSF CHI3L1 and inflammatory activity in the CNS. A decrease in CHI3L1 levels in the CSF of natalizumab-treated MS patients was shown compared to untreated patients and IFNβ-treated MS patients [74]. However, it was also demonstrated that, despite natalizumab treatment, CHI3L1 levels remained significantly higher in RRMS patients than in healthy controls [74]. This observation suggests that inflammatory processes, including microglial activation, might not be fully inhibited by the treatment [74]. Fingolimod, a DMT with a distinct mechanism of action, appears to have a more sustained suppressive effect on CSF CHI3L1 levels than other treatments [75]. Importantly, both fingolimod and natalizumab significantly reduce CSF CHI3L1 levels in treatment-naive patients or those who received first-line DMT. However, similarly to natalizumab treatment, the fingolimod treatment of MS patients did not decrease CHI3L1 in CSF to the level of healthy controls [75].

In contrast to CSF, the effects of DMT on serum CHI3L1 appear less consistent. Studies have not found significant changes in serum CHI3L1 levels after extended treatment with IFNβ or natalizumab [109]. However, in the studies by Matute-Blanch et al., a correlation between higher serum CHI3L1 levels and a poor response to IFNβ treatment was demonstrated, and this finding confirms a potential role for serum CHI3L1 level as a predictor for response to therapy [112]. Interestingly, glatiramer acetate (GA) treatment increased serum CHI3L1 levels [112]. GA induces regulatory T cells and the shift in the Th1/Th2 balance [113]. The summary of studies regarding the effect of MS treatment on CHI3L1 levels is presented in Table 1.

### 2.5. CHI3L1 and Other Biomarkers in MS

A total of 27 articles were identified in PubMed addressing the correlation between CHI3L1 and other biomarkers found in the CSF or serum of MS patients within the last 10 years (date of PubMed search, Table 2.)

Among CSF biomarkers, NfL emerges as the most frequently reported in correlation with CHI3L1 in the analyzed studies [73,74,94,104,108,114,115,116,117]. A moderate to strong positive correlation between these two biomarkers was reported, indicating a potential correlation between CHI3L1 (glial marker = glial activation) and NfL (axonal damage marker). The available studies demonstrate a mild correlation between serum levels of CHI3L1 and NfL. However, the reported correlations of serum CHI3L1 and NfL levels are generally much weaker than correlations of CHI3L1 and NfL levels in CSF [5,105,118,119]. Elevated NfL levels in CSF and serum might reflect axonal damage induced by neuroinflammation. Therefore, the relationship between CHI3L1 and NfL is complex and most probably reflects complicated interactions between activated glial and neuron.

Sellebjerg et al. demonstrated that NfL and CHI3L1 are prognostic biomarkers in RRMS, as increased CSF levels of these biomarkers are associated with an increased risk of relapses [108]. Interestingly, Schneider et al. reported that elevated levels of CHI3L1 in the CSF of MS patients were correlated with spinal cord atrophy, but elevated NfL levels in the CSF of MS patients were correlated with brain grey matter atrophy, indicating a distinctive pathological pattern associated with each of the mentioned biomarkers [117]. Elevated CHI3L1 levels might indicate axonal loss and elevated NfL levels might indicate neuronal damage [117]. Gil-Perotin et al. observed a differentiated correlation between CSF NfL and CHI3L1 levels in MS patients depending on the clinical course of the disease [104]. High levels of NfL and low levels of CHI3L1 were found in RRMS patients, contrary to low levels of NfL and high levels of CHI3L1 in SPMS and PPMS patients [104]. A simultaneous rise in both NfL and CHI3L1 levels within RRMS patients was found to be a potential predictor of future clinical progression [104].

Although both CHI3L1 and GFAP have been linked to MS progression [30,96,118,120], their precise relationship remains unclear. A positive correlation between these two biomarkers in the CSF of RRMS [74] and serum of PPMS [121] patients has been reported. However, Abdelhak et al. failed to identify such a correlation in the CSF of PPMS patients [118]. CHI3L1 might have additional roles beyond glial activation, and its expression may not always correlate with astrocyte activation, as measured by GFAP.

The CHI3L1 CSF level has been shown to have weaker correlations with other studied CSF inflammatory biomarkers like CXCL13 and IL-6 [51,89,90,94,95].

**Table 2:** Studies on the correlation of CHI3L1 with other biomarkers in MS.

Authors, Year	Markers in Body Fluids	Study Groups	Conclusions
Huss et al., 2020 [5]	CHI3L1, GFAP, and NfL (CSF, serum)	RRMS (*n* = 47) PMS (*n* = 39) OND (*n* = 20)	– PMS: high CHI3L1 (CSF and serum) – Glia score $=\frac{CHI3L1*GFAP}{NfL}$ high in PMS CSF and serum – Serum Glia score and CHI3L1, not CSF, correlate with disability (PMS only)
Cubas-Núñez et al., 2021 [46]	NfL and CHI3L1 (CSF, serum)	RRMS (*n* = 163) PMS (*n* = 61) Controls (non-SM, *n* = 17)	– MS: high CHI3L1 and NfL – NfL high in RRMS relapses – CHI3L1 high in PMS
Hinsinger et al., 2015 [71]	527 proteins (CSF proteome analysis)	RRMS (*n* = 80) CIS (*n* = 40) PMS (*n* = 16) HC (*n* = 51)	– CHI3L1 correlates with disease progression – High CHI3L1 in CIS: rapidly converted to RRMS – Lower CHI3L2 in PMS than RRMS – CSF CHI3L1/CHI3L2 ratio discriminated PMS from RRMS
Mañé-Martínez et al., 2016 [73]	NfL, t-tau, p-tau, GFAP, S100B, CHI3L1, MCP-1, α-sAPP, β-sAPP, Aβ38, Aβ40, and Aβ42 (CSF)	CIS (*n* = 109) RRMS (*n* = 192) SPMS (*n* = 6) PPMS (*n* = 17)	– RRMS: high CSF NfL – PPMS: high CSF GFAP and CHI3L1 – CSF NfL correlated to CHI3L1 in CIS – CSF GFAP, t-tau correlated to CHI3L1 in RRMS
Novakova et al., 2017 [74]	CXCL13, CCL2, CHI3L1, GFAP, NfL, and CHIT1 (CSF)	RRMS (*n* = 59) HC (*n* = 39)	– RRMS: elevated NfL, CHI3L1, and CHIT1 – CSF CHI3L1 correlated to GFAP, NfL, CHIT1, and CXCL13
Kušnierová et al., 2020 [94]	CHI3L1, CXCL13, and NfL (CSF)	MS (*n* = 42) CIS (*n* = 14) OIND (*n* = 11) IDPNS (*n* = 4) NIND (*n* = 46) Controls (*n* = 15)	– CSF CHI3L1 correlates with NfL, not CXCL13
Gil-Perotin et al., 2019 [104]	NfL and CHI3L1 (CSF)	RRMS (*n* = 99) SPMS (*n* = 35) PPMS (*n* = 23)	– MS: high CSF NfL and CHI3L1 – RRMS: correlated CSF NfL and CHI3L1
Pomary et al., 2023 [105]	NfL and CHI3L1 (CSF, serum)	PPMS (*n* = 18) SPMS (*n* = 22) OND (*n* = 13)	– MS: serum and CSF show high NfL and CHI3L1 levels – Increased CSF CHI3L1 not linked to serum levels – No PPMS vs. SPMS difference
Lucchini et al., 2023 [107]	APRIL, BAFF, CHI3L1, CCL2, CXCL8, CXCL10, CXCL12, and CXCL138 (CSF)	RRMS (*n* = 107) PMS (*n* = 18) OIND (*n* = 10) ONIND (*n* = 15)	– Higher CHI3L1 and CXCL13 in MS vs. control – Lower CCL2, BAFF, and APRIL in RMS vs. others – High CSF biomarkers (CHI3L1, CXCL10, CXCL12, and CXCL13) are associated with CDMS risk
Sellebjerg et al., 2019 [108]	NfL and CHI3L1 (CSF)	RRMS (*n* = 109) CIS (*n* = 68)	– RRMS: high CSF NfL and CHI3L1 – CSF CHI3L1 correlated to NfL
Fino et al., 2019 [110]	BAFF, CHI3L1, sCD163, and OPN (serum, CSF)	RRMS (*n* = 46) CIS (*n* = 25) OND (*n* = 11)	– MS: high CSF CHI3L1, sCD163, and OPN – No difference in serum levels of these markers
Håkansson et al., 2017 [114]	NfL, NfH, OPN, CXCL1, CXCL8, CXCL10, CCL20, CXCL13, CCL22, MMP-9, GFAP, and CHI3L1 (CSF, serum)	RRMS (*n* = 22) CIS (*n* = 19) HC (*n* = 22)	– MS CSF: strong/moderate CHI3L1 correlations with NfL, GFAP, MMP-9, OPN, CXCL1, CXCL8, CXCL10, CXCL13, and CCL22 – MS serum: no biomarker differences
Sellebjerg et al., 2017 [115]	NfL, MBP, CHI3L1, MMP-9, and CXCL13 (CSF)	PPMS (*n* = 26) SPMS (*n* = 26) HC (*n* = 24)	– NfL, MBP, CHI3L1, MMP-9, and CXCL13 elevated in PMS – CHI3L1 correlated with NfL – Active MS: high NfL, CXCL13, MMP-9, MBP, and CHI3L1
Masvekar et al., 2021 [116]	TNFα, IL-1β, TNFβ, LIOF, TRAIL, GM-CSF, IL-10, TGFβ, IL-17F, NfL, IL-12p40, BAFF, CD14, SERPINA3, CXCL13, CD27, CD21, BCMA, and CHI3L1 (serum, CSF)	MS active/inactive (*n* = 70) HC (*n* = 5)	– Active MS: high IL-12p40, CHI3L1, TNFα, TNFβ, IL-10, and NfL – CHI3L1 correlated to IL-12p40, TNFα, TNFβ, and NfL
Schneider et al., 2021 [117]	CHI3L1 and NfL (CSF)	RRMS (*n* = 42) PMS (*n* = 89) HC (*n* = 42)	– PMS: higher CSF CHI3L1 and NfL than in RRMS – CSF CHI3L1 and NfL levels correlated
Abdelhak et al., 2019 [118]	GFAP, CHI3L1, sTREM2, and NfL (serum and CSF)	PPMS (*n* = 93)	– CSF CHI3L1 correlated to NfL and sTREM2, but not with GFAP
Fissolo et al., 2024 [121]	NfL, sGFAP, and sCHI3L1 (serum)	PPMS (*n* = 141)	– NfL and sGFAP moderately correlated – sCHI3L1 weakly correlated to NfL and sGFAP
Åkesson et al., 2023 [122]	1431 proteins (CSF and serum)	Early stage of MS (*n* = 143) HC (*n* = 43)	– CSF: elevated levels of CXCL13, LTA, FCN2, ICAM3, LY9, SLAMF7, TYMP, CHI3L1, FYB1, and TNFRSF1B in MS – Low correlations between markers
Lamancová et al., 2022 [123]	CHI3L1, sNfL, CXCL13, MCP-1, MMP-2, and MMP-9 (serum)	RRMS (*n* = 40) SPMS (*n* = 25) PPMS (*n* = 15)	– PMS: higher sNfL than RRMS and CHI3L1 increased in both with activity – RRMS: CHI3L1 correlation to CXCL13 and MCP1 – SPMS: sNfL correlation to CHI3L1 and CXCL13
Magliozzi et al., 2020 [124]	CXCL13, CCL21, IL-10, IL-12p70, CX3CL1, and CHI3L1 (CSF)	RRMS (*n* = 103) OND (*n* = 36)	– CSF IgM correlated with IL-10, CCL21, IL-12p70, CXCL1, CHI3L1, and CXCL13
Quintana et al., 2018 [125]	CHI3L1 and NfL (CSF)	MS (*n* = 51)	– Weak correlation between CSF CHI3L1 and NfL
Świderek-Matysiak et al., 2023 [126]	IFN-γ, IL-6, NfL, GFAP, CHI3L1, CXCL13, and OPN (CSF)	MS (*n* = 134) non-MS (*n* = 108)	– Elevated NfL and OPN in MS compared to NID
Pérez-Miralles et al. 2020 [127]	CHI3L1, CHI3L2, and NfL (CSF)	PPMS (*n* = 25)	– Elevated CHI3L1 and CHI3L2 – No correlation between markers
Tamam et al. 2021 [128]	Hox-B3 and CHI3L1 (CSF, serum)	CIS (*n* = 33) CIS-MS (*n* = 17) CIS-CIS (*n* = 16) RRMS (*n* = 15)	– CIS: lower CSF CHI3L1 in CIS-CIS vs. CIS-MS – Similar levels of CHI3L1 and HoxB3 in CIS-MS and RRMS
Momtazmanesh et al., 2021 [129]	NfL, t-tau, CHI3L1, GFAP, and S100B (CSF)	CIS, RRMS, and PMS, MS relapse and remission, (*n* = 4071)	– NfL, GFAP, t-tau, CHI3L1, and S100B higher in MS than controls – NfL, t-tau, and CHI3L1 higher in CIS than controls – Higher CHI3L1 in MS than CIS
Ruder et al., 2022 [130]	CXCL9, GFAP, CXCL10, CXCL13, CHI3L1, and NfL (serum, CSF)	MS (*n* = 32) after hematopoietic stem cell transplantation (aHSCT)	– Two years post-aHSCT: elevated CSF GFAP and serum NfL, GFAP, and CXCL10 rise and then return to pre-aHSCT levels
Oset et al., 2023 [131]	CHI3L1, CXCL13, OPN, IL-6, GFAP, and NfL (serum)	MS (*n* = 50)	– High NfL: worse disease, relapse risk

**Abbreviations:** aHSCT: after hematopoietic stem cell transplantation; APRIL: a proliferation-inducing ligand; Aβ38: beta-amyloid 38; Aβ40: beta-amyloid 40; Aβ42: beta-amyloid 42; BAFF: B-cell-activating factor; BCMA: B-cell maturation antigen; CCL2: chemokine (C-C motif) ligand 2; CCL21: chemokine (C-C motif) ligand 21; CCL22: chemokine (C-C motif) ligand 22; CDMS: clinically defined MS; CHI3L1: chitinase 3-like protein 1; CHI3L2: chitinase 3-like protein 2; CHIT1: chitinase 1; CIS: clinically isolated syndrome; CSF: cerebrospinal fluid; CX3CL1: chemokine (C-X3-C motif) ligand 1; CXCL1: C-X-C motif chemokine ligand 1; CXCL10: C-X-C motif chemokine ligand 10; CXCL12: C-X-C motif chemokine ligand 12; CXCL13: C-X-C motif chemokine ligand 13; CXCL8: chemokine (C-C motif) ligand 8; CXCL9: C-X-C motif chemokine ligand 9; FCN2: ficolin 2; FYB1: FYN binding protein 1; GFAP: glial fibrillary acidic protein; GM-CSF: granulocyte-macrophage colony-stimulating factor; HC: healthy control; Hox-B3: Homebox B3 protein; ICAM3: intercellular adhesion molecule 3; IDPNS: inflammatory diseases of the peripheral nervous system; IFN-γ: interferon γ; IL-10: interleukin 10; IL-12p40: subunit p40 of interleukin 12; IL-12p70: subunit p70 of interleukin 12; IL-17F: interleukin 17F; IL-1β: interleukin 1β; IL-6: interleukin 6; LIF: leukemia inhibitory factor; LTA: lymphotoxin; LY9: lymphocyte antigen 9; MBP: myelin basic protein; MCP-1: monocyte chemoattractant protein 1; MMP-2: matrix metalloproteinase 2; MMP-9: matrix metalloproteinase 9; MS: multiple sclerosis; NfL: neurofilament light chain; NfH: neurofilament heavy chain; NID: non-inflammatory diseases; NIND: non-inflammatory neurological diseases; non-MS: non-multiple sclerosis: OIND: other inflammatory neurological diseases; OND: other neurological diseases; OPN: osteopontin; PCT: procalcitonin; PMS: progressive multiple sclerosis; PPMS: primary progressive multiple sclerosis; RRMS: relapsing-remitting multiple sclerosis; S100B: S100 calcium-binding protein B; sCD163: soluble CD163; SERPINA3: serpin family a member 3; SLAMF7: SLAM family member 7; SPMS: secondary progressive multiple sclerosis; sTREM2: soluble triggering receptor expressed on myeloid cells 2; TGFβ: transforming grow factor β; TNFα: tumor necrosis factor α; TNFβ: tumor necrosis factor β; TNFRSF1B: TNF receptor superfamily member 1B; TYMP: thymidine phosphorylase; α-sAPP: soluble amyloid precursor protein-α; β-sAPP: soluble amyloid precursor protein-β.

## 3. Age as a Significant Factor Affecting Biomarker Levels in MS

The interpretation of CHI3L1 levels in CSF and serum is a challenge in MS studies due to its age-related and inflammation-related concentration changes. Several studies have demonstrated age-related changes in serum CHI3L1 levels in healthy individuals [132,133,134]. Bonneh-Barkay et al. observed increased CHI3L1 concentrations in CSF in MS patients and healthy older adults compared to younger controls [45]. Several studies have confirmed this finding, demonstrating an age-related increase in CHI3L1 levels in CSF [72,104,108] and serum [34] in MS patients and healthy controls [72,135]. Additionally, a positive correlation between age and CHI3L1 levels was identified in a study of 324 patients with MS [73]. Kušnierová et al. quantified this correlation, establishing an age-based reference range for CHI3L1 in MS [94]. Burman et al. indicated the significance of age as a confounding factor [72]. In their studies, the adjustment for age eliminated the initial difference in CHI3L1 levels between types of MS (SPMS and RRMS). This suggests that age-related variations, rather than solely disease progression, might have contributed to these observations [72]. To accurately assess the relationship between CHI3L1 levels and MS disease activity or progression, statistical methods like age-adjusted partial correlations should be employed, as suggested by Canto et al. [70].

Similarly, other biomarkers such as NfL [136] and GFAP [137] exhibit a natural age-related increase. This necessitates careful age adjustment in the analysis to accurately assess the association with MS disease activity and progression. Multiple studies have highlighted the importance of age correction for NfL [135,138,139,140] and for GFAP [141]. Understanding the influence of age on CHI3L1, NfL, and GFAP levels is critical for accurately interpreting biomarker data in MS research (progression and treatment response).

## 4. CHI3L1 in MS—Not Only a Biomarker

This glycoprotein might play a complex role in the CNS under inflammatory conditions, including in MS patients. CHI3L1 has been found to be involved in the maturation of immune cells, such as dendritic cells and neutrophils, and the regulation of immune responses.

The increasing expression of CHI3L1 during dendritic cell maturation suggests a role in immune response, particularly in antigen recognition [142]. Moreover, the presence of CHI3L1 in immature neutrophils (promyelocytic HL-60 cells), and its subsequent rise during differentiation with dimethyl sulfoxide (DMSO), support the hypothesis that CHI3L1 is involved in neutrophil differentiation [143]. Studies by Kim et al. suggest that CHI3L1 might regulate the balance between lymphocyte Th1 and Th2 immune cells, potentially influencing inflammation through IFN-γ signaling [144]. Finally, the expression of CHI3L1 is higher in anti-inflammatory macrophages (M2) compared to pro-inflammatory macrophages (M1) [145].

Several studies have shown CHI3L1 involvement in neurogenesis and neuronal function, plasticity, and regeneration via engagement in the signaling pathways of neurotrophic factors [33,70,78]. This has been supported by, among others, the ability of CHI3L1 to inhibit basic fibroblast growth factor (bFGF) signaling and to suppress bFGF-induced axonal branching in hippocampal neurons [79]. Recent studies have demonstrated that CHI3L1 amplifies inflammation in the nervous system via interaction with pro-inflammatory cytokines including IL-1β, IL-6, and TNF-α [146]. However, the studies in an animal model of MS (experimental autoimmune encephalomyelitis, EAE) have revealed that CHI3L1 might also promote oligodendrogenesis (the process of generating new oligodendrocytes) by the activation of the epidermal growth factor receptor (EGFR) and the mitogen-activated protein kinase (MAPK) signaling pathway [147]. The overall effect of CHI3L1 on EAE's course has not been straightforward, as Bonneh-Barkay et al. reported a more severe course of EAE in CHI3L1 knockout mice [148], while Cantó et al. did not observe such an effect [149]. Further investigation is needed to understand the role of CHI3L1 in both EAE and MS pathophysiology. Given its involvement in multiple disease-relevant pathways, CHI3L1 emerges as a potential interesting target in MS investigation. Further studies of its specific functions and interactions with other molecules are required to develop therapeutic strategies.

## 5. Concluding Remarks and Future Perspectives

Recent investigations have highlighted the role of CHI3L1 as a promising biomarker for MS diagnosis and monitoring disease progression, as cerebrospinal fluid and serum are accessible for its measurements.

Elevated CSF levels of CHI3L1 have been associated with the disease activity, particularly predicting conversion from clinically isolated syndrome to clinically definite MS. Although its role in disease progression is less clear, several studies have shown its correlation with disease activity and severity. Despite its potential, the clinical application of CHI3L1 is limited by several factors. As CHI3L1 is a nonspecific marker, elevated in various neurological and systemic inflammatory conditions (e.g., rheumatoid arthritis, inflammatory bowel disease) and cancer, its elevated levels in MS might reflect ongoing inflammatory processes, but not MS-specific processes. Additionally, CHI3L1 levels might vary significantly between individuals, influenced by factors such as age, sex, and lifestyle. Moreover, currently, there are no standardized measurement methods, which make comparisons between different studies disputable.

To fully assess the clinical potential of CHI3L1, further research is necessary. Developing more specific and sensitive methods for measuring CHI3L1, particularly blood-based assays, could improve its diagnostic and prognostic utility. Additionally, a deeper understanding of the mechanisms underlying CHI3L1 involvement in MS pathogenesis could lead to more targeted therapeutic strategies. Ultimately, CHI3L1, when used in combination with other biomarkers, e.g., oligoclonal bands in cerebrospinal fluid, clinical assessments, and magnetic resonance imaging, might contribute to improved patient stratification and treatment decisions in MS.

## Figures and Tables

**Table 1:** Treatment effect on CHI3L1 levels.

	IFNβ	Mitoxantron	GA	Daclizumab	Natalizumab	Fingolimod	
						Switched from IFNβ	Switched from Natalizumab
**CSF**	Decrease [74]	Decrease [76]	—	Decrease [106]	Decrease [74,76,98,111]	Decrease [75]	Increase [75]
**Serum**	Increase [112] *	Without changes [76]	Increase [112] **	—	—	—	—
	Without changes [109]						

*—compared to non-responder patients according to clinical and radiological criteria; **—no difference was observed between responder and non-responder patients, and elevation was connected to GA’s mechanism, not the treatment response—no data. GA: glatiramer acetate; IFNβ: interferon-β.

## Data Availability

No new data were created or analyzed in this study.
